# Cell Communications among Microorganisms, Plants, and Animals: Origin, Evolution, and Interplays

**DOI:** 10.3390/ijms21218052

**Published:** 2020-10-28

**Authors:** Yves Combarnous, Thi Mong Diep Nguyen

**Affiliations:** 1 Physiologie de la Reproduction & des Comportements Laboratory, Centre National de la Recherche Scientifique (CNRS), Institut National de la Recherche Agronomique & Environnementale (INRAe), University of Tours, 37380 Nouzilly, France; 2 Faculty of Natural Sciences, Quy Nhon University, Quy Nhon City 55000, Binh Dinh, Vietnam; nguyenthimongdiep@qnu.edu.vn

**Keywords:** hormone, quorum sensing, receptor, bacteria, fungi, metazoa, plants, microbiota, evolution, origin of life

## Abstract

Cellular communications play pivotal roles in multi-cellular species, but they do so also in uni-cellular species. Moreover, cells communicate with each other not only within the same individual, but also with cells in other individuals belonging to the same or other species. These communications occur between two unicellular species, two multicellular species, or between unicellular and multicellular species. The molecular mechanisms involved exhibit diversity and specificity, but they share common basic features, which allow common pathways of communication between different species, often phylogenetically very distant. These interactions are possible by the high degree of conservation of the basic molecular mechanisms of interaction of many ligand–receptor pairs in evolutionary remote species. These inter-species cellular communications played crucial roles during Evolution and must have been positively selected, particularly when collectively beneficial in hostile environments. It is likely that communications between cells did not arise after their emergence, but were part of the very nature of the first cells. Synchronization of populations of non-living protocells through chemical communications may have been a mandatory step towards their emergence as populations of living cells and explain the large commonality of cell communication mechanisms among microorganisms, plants, and animals.

## 1. Introduction

The cell is the structural and functional unit of all living organisms. Unicellular organisms such as bacteria, archeas, yeasts, or protists consist of a single cell. In contrast, multicellular organisms such as sponges, nematodes, trees, or vertebrates can comprise from a few hundred to several billion or even trillion of cells. In such complex multicellular species, the cells exhibit many differentiated phenotypes playing highly-specialized functions and are often associated within individualized organs. The cellular activities are coordinated at the level of each organ as well as between organs, in order to allow adaptation of the living organism as a whole to its environment. This coordination rests on the traffic of information between all cell types and constitutes in animals the endocrine, nervous, and immune systems.

Most intercellular mediators are either small, simple molecules, such as nitrogen oxide (NO), ethylene, nucleotides (ATP, AMP), and amino acid derivatives (serotonin, melatonin, auxin, thyroxine, homoserine-lactones, etc.), or amino acid polymers (peptide, protein and glycoprotein hormones, cytokines, growth factors, etc.), lipid derivatives (steroid hormones, prostaglandins, jasmonate, etc.), and various other molecules.

In unicellular species, all cells seem identical and independent, suggesting that it is the survival and reproduction of each of them that ensures the sustainability of these species. Nevertheless, communications do indeed exist between these cells, hence allowing some coordinated common responses as well as specialized roles for sub-populations. These coordinations optimize the development and survival of unicellular species populations.

Moreover, information exchanges are not limited to cells inside an organism, not even to cells belonging to one given species. They also exist between cells from different species, whether unicellular or multicellular.

Unicellular species have been prosperous for approximately 3.5 billion years and still represent the vast majority of living species. They can communicate indirectly through soluble mediators to regulate their growth and/or phenotype according to the available food resources. In spite of this success, multicellular species relying on direct cellular adhesiveness and specialization also emerged several hundred million years ago. The main innovation in plants and metazoan is the formation of highly specialized cell populations, requiring proper communications inside these organisms to ensure their development, survival, and reproduction.

All intercellular communications rely on intercellular messengers (mediators) and their cognate receptors in their target cells. The receptors play pivotal roles as they connect intercellular communications to downstream intracellular signaling. Despite the considerable diversity of communications among living species, the number of receptor types and transduction mechanisms is somewhat limited, suggesting their conservation during the Evolution.

Cell communications occur not only inside multicellular species, but also in unicellular species, as well as between different species, whether unicellular and/or multicellular.

## 2. Cell Communications and Communitarianism in Unicellular Species

By definition, unicellular species do not possess specialized differentiated cells and organs. Nevertheless, communications exist between cells of unicellulars, inside or between different species.

### 2.1. Bacteria

The membrane receptors in bacteria often directly respond to nutrients [1], or to quorum sensing (QS) signaling molecules [2], or to various other molecules in the environment. Bacteria also directly interact with each other, in particular in the soils [3] or in animal guts or at the level of plant roots.

Many bacteria communities develop in synchronized fashion to face changing environments [4] and form biofilms in which they share nutrients and protection [5].

The establishment of a biofilm requires a sufficient number of bacteria, and many QS signaling molecules (autoinducers) and cognate receptors exist in various bacteria [6,7]. Four main general types of autoinducers (AI) have been described: (1) AI-1, mainly present in Gram-negative bacteria, are *N*-acylated homoserine-lactones (AHLs) with a core homoserine-lactone ring and a 4- to 18-carbon acyl chain with eventual modifications [8]. The AHLs bind to specific LuxR-type cytoplasmic receptors [9], which control transcription of numerous virulence genes, and to LuxN-type membrane receptors. (2) AI-2, present in both Gram-positive and Gram-negative bacteria, are furanosyl borate diesters, considered as universal signal involved in unicellular interspecies communications [10,11]. (3) AI-3, mainly in Gram-positive systems, typically use secreted oligopeptides [12,13] and two-component systems (TCSs), consisting of membrane-bound sensor kinase receptors (QseC) and cytoplasmic transcription factors (QseB) that direct alterations in gene expression [2,14,15,16]. (4) PQS (pseudomonas quinolone signal) makes use of 2-heptyl-3-hydroxy-4(1H)-quinolone [17], which binds to its specific LysR-type transcriptional regulator receptor (PqsR) to control the synthesis of a rhamnolipid, which is a critical surfactant for biofilm formation [18].

Bacteria pump ions across their membranes, and several recent papers have reported spikes of electrical activity in bacteria, which suggest that, like neurons, bacteria use potassium ions to propagate electrical signals [19]. This electrical communication is considerably quicker and more extensive than QS.

Viruses and other capsidless replicons such as plasmids, transposons, and viroids promote an enormous amount of exchanges among bacteria. Bacteriophages are present everywhere bacteria are located and kill many of them, but never all of them, without the risk of disappearing themselves. In a way, bacteriophages are kind of QS mediators, allowing bacteria to sense how many of them have already succumbed. For example, the coordination of the lysis-lysogeny decision of phages in *Bacillus* is based on the release of phage-specific communication hexapeptides. These peptides are imported in bacteria by the oligopeptide permease transporter (OPP) and bind to their intracellular receptors, which then no longer activate the expression of an inhibitor of lysogeny [20,21,22].

### 2.2. Yeasts and Fungi

Direct communications between yeasts occur through membrane proteins such as flocculins, which are lectins recognizing their partners’ polysaccharide chains to form a solid mass (veil, biofilm) [23,24]. Yeasts also communicate via soluble molecules that can diffuse and affect the community’s organization in the long term by quorum sensing. QS molecules identified in fungi include peptide pheromones, oxylipins, aromatic alcohols (such as tyrosol and farnesol [25]), α1–3 glucans [26], and pantothenic acid [23,27,28]. A number of fungi among Arthoniomycetes also participate in the formation of lichens, and thus do communicate with algaes (see 1.4).

### 2.3. Large Unicellular Eukaryotic Microorganisms (Formerly Called “Protists”)

Amoebas, paramecia, or trypanosomes present, in the case of limited resources, phases with several different phenotypes. In the amoeba, *D. discoideum* cyclic AMP acts as an intercellular mediator. Its export co-occurs with its intracellular synthesis by adenylate cyclase, which contains a general structure similar to ATP binding cassette (ABC) transporters [29], known to export anionic cargoes like cAMP. ABC transporter inhibitors disrupt amoeba development in a manner consistent with a lack of cAMP export, indicating that cAMP plays an intercellular messenger role during *D. discoideum* development [30]. When the density of bacterial prey becomes low compared with the amoeba population, the concentration of the pre-starvation factor (PSF) sensor protein decreases, leading to activation of protein kinase YakA, which relieves the inhibition of expression of a cAMP-dependent protein kinase. The latter stimulates the expression of several genes, including a cAMP membrane receptor responsible for the aggregation in spores through a positive cAMP-dependent activation loop [30,31]. Amoebas also use quorum sensing-like communication systems based on the complex dipeptide glorin [32] to coordinate the periodic transition from uni- to multi-cellularity.

Among trypanosomes, the cross-species interactions between QS systems have important implications for their virulence, transmission, competition, and evolution [33]. The parasites exploit oligopeptide signals generated by released peptidases to monitor cell density. Then, a transporter protein takes up combinations of small oligopeptides to control trypanosome differentiation either towards the actively dividing slender form or towards the stumpy non-dividing form [34].

### 2.4. Lichens

Lichens are long-term intimate symbiotic partnerships between mushrooms (Arthoniomycetes) and photosynthetic algaes occupying nutrient-poor niches [35]. Inter-species communications are thus crucial for the initial steps of symbiosis in lichen formation and development [36]. Recognition of compatible algal cells is performed by specific lectins produced and secreted by the potential mycobiont. For example, the lectin of *Peltigera canina* recruits both algal cells (chlorobionts) and cyanobacteria (cyanobionts), forming high-affinity bonds with different galactose units in the poly-α-1,4-galactoside side chain of their wall. Free non-motile cells of the cyanobacterium that bind the lectin are recruited and move toward the lectin source [37,38]. Upon reaching the maximal concentration of the gradient, the cells become desensitized, and lectin binding promotes stable cell aggregation between the two partners [39]. Bacterial communities (particularly cyanobacteria) also participate in the constitution of these inter-species associations [40]. Some of these bacteria possess genes allowing the synthesis of auxins, and can thus attract their eukaryote partners (mushrooms and/or algaes).

A number of bacteria and archaea live in even harsher environments and, in a similar way, associate with other species to withstand extreme temperature, pressure, dryness, salinity, pH, or other chemical conditions. These associations require intra- and inter-species cellular communications, not simply mutual metabolic complementation [41,42,43].

## 3. Cell Communications in Multicellular Species

Multicellularity has only emerged and succeeded in fungi, algae, plants, and animals [44]. All multicellular organisms are eukaryotes, and the nuclear chromatin structure in each cell controls its specific fate through the expression of homeotic genes. This development in animals is controlled and coordinated only by a few extracellular ligands (such as Hh, Wnt, FGF, BMP, and some others) providing complex structuring information via their identities, concentrations, combinations, and dynamics [45], as well as via the constitution of niches, particularly for stem cells [46,47]. Intercellular communications through soluble mediators already existed in unicellular organisms, probably for 3.5 billion years. The specialization of various cell populations in multicellular species emerged much more recently, approximately 600 million years ago [48]. In these species, both direct and indirect cellular communication mechanisms co-exist.

Intercellular communications in multicellular organisms arise via four different molecular mechanisms: (1) cytoplasmic bridges, (2) exosomes and ectosomes, (3) direct interactions between membrane proteins of adjacent cells, and (4) soluble messenger molecules (mediators) controlling more or less distant target cells.

### 3.1. Communications through Direct Cytoplasmic or Membrane Contacts

There are several types of cytoplasmic bridges, including gap junctions [49,50], plasmodesmata [51,52], tunneling nanotubes [53,54], and others formed by incomplete cytokinesis [55,56,57,58,59,60]. Exosomes and ectosomes are two distinct kinds of extracellular vesicles (EVs) generated by all types of cells [61], particularly at the tip of primary cilia [62]. Their cargoes include proteins, lipids, and nucleic acids, which are imported into the target cells’ cytoplasm to affect the activity of transcription factors; signaling proteins; and many enzymes in animals, plants, and microorganisms [61,63,64,65].

Many membrane multidomain proteins [66] allow cell–cell adhesiveness and communications. Most of these proteins exhibit repeated stable conformations [67,68,69,70,71,72,73]. In animals, cells are also in contact through the extracellular matrix (ECM), kind of an extracellular “glue”, which includes proteins such as collagens, fibronectins, or laminins [74,75,76], as well as oligosaccharides [77] interacting with cell membrane cadherins, integrins, CAM (cell adhesion molecules), selectins, and so on [78,79,80]. In plants, there is a thick polysaccharide wall around most cells, which prevents the plasma membranes of neighboring cells from coming into contact, so that germ cells require a particular structure, the pollen tube, to come in contact [81].

### 3.2. Communications through Soluble Mediators

Almost all cells, in uni- and multicellular organisms, produce soluble messenger molecules (mediators), capable of influencing distant cells. In animals, these mediators include hormones, neuromediators, cytokines, growth factors, morphogens, and so on. Their target cells can contact the mediator-emitting cell (post-synaptic cells, immune cells) or be at a considerable distance in another individual (target cells for pheromones). In plants, many different hormones also exist and act at a very short distance, such as ethylene, or at distance such as auxin, gibberellins, florigen, and various phyto-œstrogens.

The mediators’ receptors are located at the functional interface between intercellular communications and intracellular signaling. They belong to two prominent families: (1) membrane receptors with their binding site at the external surface of cells, and (2) intracellular receptors acting at the level of DNA (nuclear receptors in eukaryotes). The receptors of the first group bind to mediators that do not penetrate the cell, whereas those of the second group perceive only ligands capable of penetrating the cells.

The mediators’ receptors in plants and animals are either soluble intracellular transcription factors or proteins inserted in the plasma membrane (Table 1). The nuclear receptors exist in both animals and plants, but, whereas they form a large family of related transcription factors in animals [82], in plants, diverse proteins serve as intermediaries in the genomic actions of the hormones [83]. These binding proteins in plants are structurally very diverse, in contrast to the kinship of animals’ nuclear receptors. Nevertheless, the general mechanisms in animals and plants appear to share many similarities [84].

**Figure 1:**
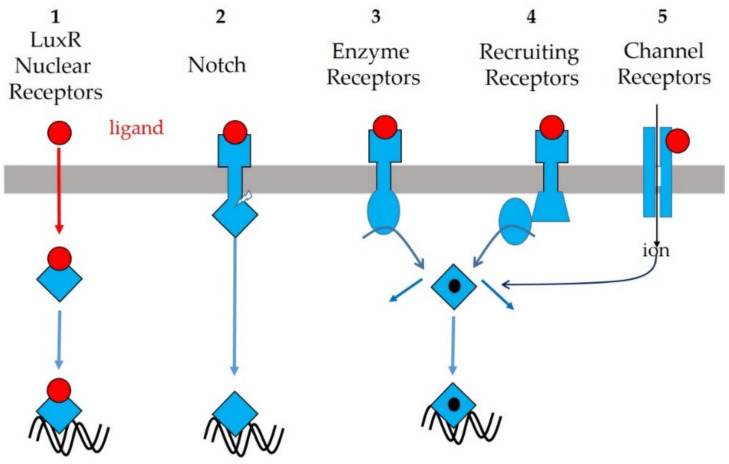
General view of intercellular messengers’ receptors and their downstream signaling pathways. (1) Intracellular ligand-regulated transcription factor. (2)–(5) Plasma membrane receptors: (2) protease-cleavable receptor with intracellular domain exhibiting transcriptional activity; (3) enzyme receptors (Tyr, Ser/Thr, His kinases, GMPcyclase, phosphatase); (4) non-enzymatic receptors recruiting cytoplasmic partners (kinases, G proteins, scaffolding proteins); and (5) channel receptors (ionotropic). For details, see also Table 1.

In brief, as shown in Figure 1, the plasma membrane receptors of animals and plants are either

1. channel-receptors letting in specific ions [85,86];
2. receptors acting by direct catalysis (receptors with intrinsic enzymatic activity, i.e., protein kinase activity [87,88], or phosphatase activity [89], or guanylate cyclase activity [90,91]);
3. receptors acting through recruitment of various downstream intracellular effectors (G proteins [92,93,94,95,96,97,98,99], adenylate cyclases [100,101,102], phospholipases C [103,104,105], soluble protein kinases [106,107,108,109,110,111], methyltransferases [112], proteases [113,114,115,116], and so on).

These receptors possess (a) either a very short or huge extracellular domain (ECD), (b) one or several membrane-spanning sequences (seven in G protein-coupled receptor (GPCR)), and (c) an intracellular domain (ICD) comprising one or several peptide sequences. The ECD permits ligand binding, whereas the ICD either possesses enzymatic activity like the insulin receptor (tyr-kinase) or TGFβ receptor (ser/thr kinase) (Figure 1); or recruits cytoplasmic soluble enzyme(s), like the growth hormone and cytokine receptors (Jak and Tyk kinases); or recruits G-proteins, like the numerous GPCRs (Figure 1); or is clipped off as a transcription factor to perform intracellular signaling, like the Notch receptor (Figure 1). Membrane receptors and downstream partners are generally concentrated at the level of lipid rafts [117] or primary cilium in metazoan [62,118]. The downstream signaling pathways and their evolution have been thoroughly described in a recent comprehensive review [119].

## 4. Cell Communications between Uni- and Multi-Cellular Organisms (Microbiotas)

Microbiotas are sets of commensal microorganisms of animals or plants occupying a favorable environment and exchanging numerous advantages to their shared host. It is most likely that these associations existed right from the origin of plant and metazoan lineages.

### 4.1. In Animals

Animals host microbiotas at different locations in their body. In vertebrates, microbes mainly settle in their intestine as a complex community (10–100 times the number of the host’s own cells), comprising aerobic and anaerobic bacteria, archeas, viruses, fungi, and other microbial eukaryotes, playing critical roles in the host physiology. Most insect guts contain relatively few microbial species compared with mammals, but some harbor large communities of specialized bacteria. The microbiota in animals have significant influences on endocrine, nervous, and immune systems.

The communications of microbiota bacteria with animal host cells affect not only local intestinal functions [120,121], but also general integrated functions. The best-known bacterial recognition patterns are lipopolysaccharide (LPS) and peptidoglycan (PG), which act through binding with the host’s pattern recognition receptors (PRRs), including Toll (*Drosophila),* Toll-like receptors (TLRs) (vertebrates), and C-type lectin receptors (CLRs) (vertebrates). In vertebrates, membrane TLRs of the epithelial and lymphoid cells of the small intestine, which are responsible for innate intestinal immunity, differentiate microbiota microorganisms and promote immunological tolerance towards them [122]. Moreover, chemical mimicry of animals’ signaling molecules such as neuromediators or hormones can be present in commensal bacteria. For example, *N*-acyl amide synthase in commensal bacteria produce lipids, such as endocannabinoids, which interact with GPCRs involved in gastrointestinal tract physiology [123]. The mammalian gut microbiota affects not only the host’s immune system, but also its autonomous [124,125] and central [126,127] nervous systems, in particular, via the vagus nerve from the intestine towards the hypothalamus, where it controls the host’s appetite [128] and other behaviors [129].

Fungal microbiota secretes substances such as pyrazines, which play very important roles in animals’ physiology and behavior. Some fungal pyrazines are used as a guide for individuals to find their way back to the anthill. In the model species *Drosophila*, behavioral responses to 25 fungal pyrazines vary widely despite their chemical similarity, ranging from strong, attractive responses to no response at all. Two olfactory receptors in *Drosophila*, Or33b and Or59a, yield remarkably long-lasting responses to certain pyrazines [130].

### 4.2. In Plants

The conquest of land by terrestrial plants occurred thanks to their interactions with fungi, and the survival of animals and plants is dependent on their respective microbiota. It is thus interesting to consider which molecular communication tools are present in unicellular and/or multicellular species, and how these mechanisms ensure optimal cell interactions favoring their respective survival and joint expansion.

The rhizobiota (root microbiota) is the set of the soil organisms (bacteria, mushrooms, virus, and so on) interacting with plant roots and thus supplying them with many compounds that they do not synthesize. Plants contain a significant number of pattern recognition receptors (PRRs) that share remarkable structural and functional similarity with the Drosophila Toll receptor and mammalian Toll-like receptors (TLRs) recognizing various pathogens or mediators. The plant PPRs are either membrane receptors with intrinsic intracytoplasmic kinase activity (RLKs) or non-kinase membrane receptors (RLPs) able to recruit RLKs and cytoplasmic kinases [131,132]. Plants are also able to cope with their microbiota through the emission of extracellular vesicles [64] and complex cross-talks through their respective secretomes to constitute functional holobionts [43,133].

Mycorrhiza (symbiosis of fungi with plant roots) is considered to be at the origin of the colonization of dry land by water plants, approximately 450 million years ago. More than 90% of the living land plants can form a mycorrhizal symbiosis, and non-mycorrhizal status is an exception. Besides, the mycorrhizal network allows communications between plants of the same species or different species. There are two types of mycorrhization: arbuscular mycorrhizae (AM) and ectomycorrhizae (EM).

The arbuscular mycorrhizal (AM) fungi have developed a symbiotic relationship with most land plants, facilitating the uptake of minerals and water from the soil by plants [134,135] and providing carbon sources to fungi. Hormones from the plants play a prominent role in AM establishment [136]. In AM, fungal hyphae penetrate the cortical tissues of roots, between the cell wall and plasma membrane, thus coming into close contact with plant cells. Although AM interactions are physically restricted to the roots, they influence the whole-plant performance [137].

The ectomycorrhizae (EM) develops a fungal system close to the roots with a mantle surrounding short roots and a network (called Hartig net) that penetrates between the roots’ cortical cells.

The establishment of AM or EM requires complex dialogue between the two partners, including the perception by roots’ His-kinase receptors of specific lipo-chitooligosaccharides (LCOs), called Myc-LCO, secreted by fungi. These receptors share a high degree of similarity with receptors for plant hormones (ethylene and cytokinin) and must have played an essential role in their interaction with plants, leading to their joint conquest of land.

Endophytic bacteria represent a major part of the plant microbiota, which exert growth-promoting activities by increasing the availability of limiting plant nutrients, such as nitrogen, iron, and phosphorus [138,139], and providing photosynthetic carbon to bacteria through the formation of nodules [140]. The first signal comes from the roots and is a cocktail of flavonoids, which stimulates the synthesis of nodulation (Nod) factors by the bacteria, which cause the formation of nodules around them by roots. Nod factors are lipo-chitooligosaccharides (LCOs) that act via the hetero-dimerization of root membrane receptor-kinases and a calcium- and calmodulin-dependent kinase (CCaMK) [141,142]. Ethylene signaling is not required for Nod factor signaling, infection development, or nodule organogenesis, but it is for initiation of nitrogen fixation and negative regulation of nodulation [143,144,145]. In return, ethylene production by the plant is modulated by Nod factors [146]. Gibberellins exert both positive and negative effects on nodulation because they act as suppressors of infection, but also as promoters of nodule organogenesis. Gibberellins and ethylene act through independent pathways in nodule development [147]. Cytokinins also interact with the previous phytohormones in different ways to impact nodulation [148].

## 5. Cell Communications between Different Multi-Cellular Organisms

### 5.1. Cell Communications between Animals

In the present paper, we consider cell communications as integrated networks leading to a mutually beneficial equilibrium for two communicating species. Therefore, the detection of their hosts by parasites cannot be considered as communication. Pheromones are very important volatile mediators inside each animal species, and have been introduced before. Although involved in inter-species detection, kairomones cannot be considered as communication molecules as they benefit only parasites for detecting potential hosts, and not for establishing a mutual favorable interaction.

### 5.2. Cell Communications between Plants

Plant volatile organic compounds (VOCs) such as sesquiterpenes, emitted particularly under salicylic and jasmonic acid control [149,150], are major vehicles of alarm information between plants in response to herbivore damage. VOCs may either directly affect cell membranes’ potentials and induce endogenous signal transduction cascades, or enter the cell and directly bind to co-repressors of stress-responsive genes.

Plants can integrate multiple volatile cues into specific adaptive defense responses [151]. It is interesting to point out that these communication channels can be private for one plant species or even one genotype inside a species. By contrast, others are open channels, allowing information sharing with different species [152]. This sharing may be favorable despite the cost of alarm signaling toward potential competitor species [153]. Indeed, it allows cooperation in herbivore insect exclusion from their common neighborhood through herbivore-induced plant volatile molecules acting via epigenetic mechanisms that sustain the memorization of the defense response [154].

### 5.3. Cell Communications between Animals and Plants

Plants broadcast signals either to attract or to repel animals (pollinating and herbivore animals, respectively). For pollination, most of the visitor species interact with only one or very few plant species. Mutualistic interactions imply sophisticated interplay of floral stimuli (scent, color) and the sensory systems of pollinators (bees, hawkmoths, geckos, and so on), among which vision and temperature play significant roles. Information transfer between plant species reduces the attraction of bee pollinators to herbivore-attacked plants or warned plants relative to undamaged/unwarned plants [155]. When wounded by herbivore insects, some plants, like tomatoes, produce the 18aa peptide systemin, which binds to its receptor and stimulates the jasmonic acid cascade to produce bad-tasting chemicals and escape further consumption [156].

Cell communications from animals to plants are not a very usual research field. Nevertheless, it has been recently reported that bees bite plants’ leaves to induce their flowering to get access to pollen and chemicals in the insects’ saliva may be involved [157]. For the time being, the precise molecular and cellular mechanisms are not known.

## 6. Origin and Evolution of Cell Communications

The origin and evolution of cell communications obviously cannot be directly studied experimentally. Only a global view of current cell communications can guide us to speculate about their origin and evolution.

### 6.1. Origin

The origin of inter-cellular communications must be intricated with that of the cells themselves. Life did not emerge directly as cellular organisms, but initially as simple genetic or chemical replicators [158,159,160,161]. The dynamic kinetic stability hypothesis that bridges abiogenesis to life [162] would require sustained communications to maintain the required *far-from-equilibrium* state during this process, whatever it was precisely. Even if life could have begun as a system of such RNA and polypeptide replicators, chemically occurring membranes must have played a primary role in the very first steps of life on Earth. Indeed, compartmentalization by membranes has led to cellularity [163] that is today the hallmark of all living beings.

Primordial ancestral membranes likely were single-chain amphiphiles that formed vesicles and protocells, and gradually evolved into phospholipids during the emergence of cells [164]. Membranes not only enclose a limited volume where genetic and metabolic reactions can occur much more efficiently, but also provide the site for chemical potential energy in the form of the proton-driven ATP synthase motor [165,166].

It is highly challenging to trace back the unique history of life, approximately 3.8 billion years ago [167]. A wide range of theoretical and experimental methods have been developed for constructing and testing possible evolutionary scenarios of prebiotic evolution, towards the emergence of the first living cell(s) [168,169]. It is generally accepted that multicellular organisms emerged from unicellular organisms [170] and that communications became mandatory only a long time after cell emergence. However, cyanobacteria, one of the earliest types of bacteria, dating back to between 3.4 and 2.8 billion years ago, formed huge colonies (stromatolites) [171]. It is thus likely that life actually began much earlier, perhaps as early as 3.8 billion years ago, and most likely under the form of synchronized communicating cell populations.

Taking this into consideration, the protocells and first living cells must have never existed in a truly isolated state. Chemical communications might have already existed among protocells and were probably essential for the emergence of living cells, as suggested by the dynamic kinetic stability hypothesis [162]. If true, this hypothesis implies that the first living cells were not isolated and emerged from inanimate protocell communities without rupture of communications between them.

Compartmentalization of the initial chemical “primordial soup” by membranes might have favored the interactions between polynucleotides and polypeptides to initiate the very first steps leading to metabolism and heredity. Cross-communications between the proto-cells must have played a favorable role in the emergence of genuine cells (prokaryotes and eukaryotes).

**Figure 2:**
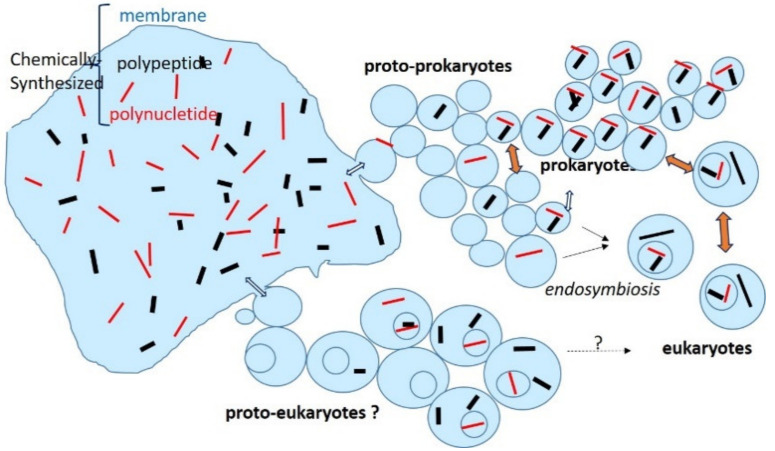
Possible early origin of communications between proto-prokaryotes, prokaryotes, eukaryotes, and hypothetical proto-eukaryotes.

The hypothesis of synchronized protocell populations is difficult to prove (or disprove), but would make sense. For example, to avoid extinction by dilution, the protocells must out-compete other vesicles either by having a more rapid cycle, thereby generating more progeny during division, or by surviving destructive processes more efficiently [172]. Synchronization of populations of protocells, requiring chemical communications, should have given them an advantage in terms of number and then facilitated their emergence into living cell populations. These first cell populations, retaining chemical communications, would have offered a more protective and stable milieu for the emergence of life. The emergence of prokaryote as well as eukaryote cells from a common (proto-karyotic) syncytial ancestral root [173] is a stimulating view in this respect (Figure 2). Nevertheless, it is much more generally accepted that eukaryogenesis occurred only around 1.7 Gy ago by endosymbiosis involving archaea and bacteria [174,175], most likely with no arrest in their previous communication networks and, therefore, is the very beginning of communications between prokaryote and eukaryote cells.

The fact that living cells are non-equilibrium systems suggests that life can emerge only from non-equilibrium chemical systems, thus needing energy input. The energy from the sun is the ultimate source of energy for sustaining life on Earth. From an astrobiological standpoint, non-equilibrium chemical systems can arise naturally when solar irradiation strikes rotating surfaces of planets [176]. It is tempting to imagine that solar photoperiodism might have synchronized protocells’ chemical functioning, and then kept synchronizing biological metabolism and divisions in the newly-formed cell populations. Their synchronization should have favored the functional cohesion between cells through circadian light exposure. Interestingly, cell communications are still synchronized by light, circadian rhythms in a vast number of prokaryote, plant, and animal species.

### 6.2. Evolution

Intercellular mediators emerged during Evolution in conjunction with the appearance of their cognate receptors. In some cases, one of them (ligand or receptor) appeared first and “found” its counterpart later, but, more often, they appeared “simultaneously” at the phylogenetic time scale [177]. It is likely that molecules with no partner (i.e., with no role) are not conserved for long in the following generations. When a ligand or receptor appeared a long time before its current partner, it likely had another interacting partner (i.e., another role) in the meantime.

The most ancient intercellular messengers were probably either intracellular metabolites like ATP and other nucleotides, released by damaged cells, or naturally excreted metabolites. In the receiving cells, their initial targets were probably the same proteins as those recognizing their proper internal molecule. To discriminate the incoming messenger molecules from their own intracellular ligands, part of these intracellular proteins must have evolved to become located at the surface of the cells. The plasma membrane is the most favorable place for such receptors to discriminate outside messengers from their own metabolites. De novo protein domains emerging from non-coding thymine-rich DNA sequences must have played advantageous roles in this evolutive process. Indeed, such DNA sequences exhibit a high potential to be translated into transmembrane domains [178]. These domains next to the coding sequences of copies of the intracellular protein have led to this new location at the plasma membrane. Because these cells belong to the same species, this initial phenomenon corresponded to an autocrine mechanism.

From this simple autocrine communication, diversification of cell phenotypes has led to more and more complex and specific communication networks in microorganisms, plants, and animals.

In prokaryote and eukaryote microorganisms, quorum sensing represents the most general example of distant intercellular communications. In plants, specialized molecules play specific roles in communications between their various parts for coordinating their development and functions. In animals, an even higher integration occurs through their endocrine, neuronal, and immune systems. The neuronal system is unique to animals, but the ion channels and proteins involved in synapses originated long before their emergence. Moreover, the animals with a nervous system do not form a monophyletic group. Therefore, the extension and disappearance of membrane ion-channel genes during Evolution can explain the differences in the functioning of the nervous systems in these various species [179].

Cell communications generally require highly specific ligand–receptor high-affinity binding. High specificity necessitates that the receptors are strictly specific, and do not bind molecules with structures close to that of their ligands. For example, steroid hormone receptors discriminate each hormone, and so do the glycoprotein hormone receptors, despite that these hormones share a common alpha-subunit, which plays an essential role in receptor binding [180]. The “negative specificity” hypothesis that we have previously introduced [181] postulates that high affinity and high specificity can be brought by different parts in the ligand–receptor interfaces. During Evolution, more and more numerous and specific ligand–receptor pairs have appeared, requiring that each receptor of course recognizes its own ligand, but also cannot be activated by a molecule akin to it. Therefore, each receptor during Evolution was shaped to bind its own ligand and to reject structurally-related molecules, particularly ligands for other receptors. It is interesting to point out that the estrogenic receptor that does not bind other steroid hormones (cortisol, corticosterone, progesterone, testosterone) is activated, although weakly, by a myriad of organic molecules from industrial origin that do not resemble estradiol (bisphenols, phtalates, and so on) and are known as endocrine disrupter compounds. No specific receptor-binding inhibition against these molecules could be selected during evolution as they did not exist [182].

Cell communications are based on molecules and mechanisms that appeared at the very early steps of life emergence, thus probably at the same time as cells themselves. During Evolution, a huge diversification of ligand–receptor pairs occurs with high conservation to retain high affinity and modifications of details to gain specificity through inhibition of binding of the wrong ligands.

### 6.3. Interplays

Cell communications show considerable diversification, allowing outstanding cell interactions inside multicellular species, but also between different species, either uni- and/or multi-cellular. In animals, various physiological systems have emerged (endocrine, neural, immune), which share common molecular mechanisms that ensure their close functional integration in terms of biological and behavioral responses [183,184,185,186]. In the most “primitive” metazoa, the distinction between neuronal, endocrine, and immune systems is not clear-cut. For example, in cnidarians (medusa, corals), there is no endocrine gland, but the diffuse neurons secrete neuromediators and molecules that are chemically related to vertebrate hormones such as steroids, melatonin, or GnRH [187]. During Evolution, the endocrine system has gained in complexity in all invertebrates, but even more in vertebrates because of the dual duplication of the entire genome at the root of vertebrates’ radiation [188]. This has permitted the emergence of the pituitary as a major integrator of endocrine and neuro-endocrine functions [189,190,191]. In individual plants, the physiological integration relies on less complex morphological and functional integration than in animals. However, because of their immobility, plants establish numerous stable interactions with soil fungi and bacteria. During Evolution, the root–nodule symbiosis concerned more numerous species than today [192], indicating its loss in multiple clades. Conserved co-regulated genes found within legumes paved the way for nodule formation and nitrogen fixation [193]. The ability of bacteria to decrease plant ethylene levels by the expression of the enzyme 1-aminocyclopropane-1-carboxylate (ACC) deaminase or via the production of rhizobitoxine was found to be essential for leguminous plants to nodulate [194]. Mycorrhization preceded bacterial symbioses during the conquest of land by terrestrial plants [195], thus showing tripartite interactions in this process during Evolution. Through their microbiota, individual plants can communicate with others, of the same or other species, leading to even higher integration [196] thanks to the shared molecular language of plants, fungi, and bacteria..

## 7. Conclusions

Cellular communications play pivotal roles in all uni- and multi-cellular species, leading to an outstanding variety of essential biological processes not only inside each species, but also for numerous favorable interactions between uni- and multi-cellular species. Inter-species communications were selected during Evolution when mutually beneficial. They have been made possible by the high degree of conservation of the basic mechanisms of ligand–receptor pairs in evolutionary remote species.

## Figures and Tables

**Table 1:** Intracellular and membrane receptor families in microorganisms, plants, and animals. GPCR, G protein-coupled receptor; AHL, N-Acyl homoserine lactone; NO, nitrogen oxide; TLR, toll-like receptor; QS, quorum sensing; PQS, pseudomonas quinolone signal; LPS, lipopolysaccharide.

Receptors		Mechanisms	Ligands	
Intracellular, Ligand-Regulated, Transcription Factors	LuxR (LasR, TraR)	Transcription	QS autoinducers (various AHL)	Bacteria
	LysR (PqsR)	Transcription	PQS (various Quinolones)	Bacteria
	Nuclear Receptors	Transcription	oleate ergosterol	Fungi
		Transcription	Florigen (PEBP)	Plants
		Transcription	brassinosteroids, gibberellins, jasmonates, salicylates	Plants
		Transcription	Steroid and thyroid hormones, VitD, RA, prostaglandins	Animals
Other, Intracellular, Ligand-Regulated Targets	Ubiquitin-ligase	Protein degradation	auxin	Plants
	Monomeric G protein (Ste2–3p)	?	farnesol tyrosol tryptophol	Fungi
	NO sensing protein	Two-step His/Asp transfer	Nitric Oxide	Bacteria
	soluble guanylate cyclase	cGMP increase	Nitric Oxide	Animals
	His kinases	Two-step His/Asp transfer	Ethylene, brassinosteroids	Plants
	His kinases	Two-step His/Asp transfer	arabinose, Mg++	Fungi
	Di-guanylate cyclase	di-cGMP increase	environment signals	Bacteria
	Tyr kinases (RTK)	IRS, Shc Tyr phosphorylation	IGF, insulin, EGF	Animals
	Ser/Thr kinases	SMAD S/T phosphorylation	TGFβ, BMP, Activin, Inhibin	Animals
	guanylate-cyclase	cGMP increase	ANF	Animals
	Tyr-phosphatase	Tyr dephosphorylation	Proteoglycans or unknowns	Animals
Plasma Membrane, Non-Enzyme, Receptors	ionotropic R	Ion entry	glutamate acetylcholine, amino acids?, mechano-stress, sterols	Animals, Plants, Bacteria
	Notch	Transcription domain liberation by proteolysis	Cell membranes proteins (Delta Jagged Serrate)	Animals
	Cytokine R	Kinase recruitment (JAK)	GH, Prl, interleukins,	Animals
	BcR, TcR, FcR	Kinase recruitment (lck, lyn)	MHC, antigens, immunoglobulins	Animals
	TNFR	TRADD TRAF RIP caspases recruitment	TNF	Animals
	Integrins	SFK Talin Kindin Vinculin recruitment (cytoskeleton organization)	Extracellular matrix components	Animals
	Toll, TLR	Myd88 recruitment	LPS, bact DNA, flagelin	Animals
	7TMR (GPCR)	Trimeric G-protein recruitment	alpha mating factor	Yeast
	7TMR (GPCR)	Trimeric G-protein recruitment	hormones neuromediators, pheromones	Animals
	7TMR (mGluR), 7TMR (Frizzled)	Homer recruitment, Dishevelled recruitment	Glutamate, Wnt	Animals

## Abbreviations

ABC	ATP binding cassette
AHL	N-Acyl homoserine lactones
AI-2	autoinducer-2
AI-3	autoinducer-3
AM	arbuscular mycorrhizae
AMP^(1)^	antimicrobial peptide
AMP^(2)^	5’-adenosine mono-phosphate
ATP	5’-adenosine tri-phosphate
BMP	bone morphogenic protein
CAM	cell adhesion molecule
cAMP	3’5’cyclic-AMP
CBP	CREB binding protein
CLR	C-type lectin receptor
CREB	cAMP-responsive element binding protein
CRP	cyclic-AMP receptor protein
DAMP	damage-associated molecular pattern
ECD	extracellular domain
eDNA	extracellular DNA
EM	ectomycorrhizae
EV	extracellular vesicle
FGF	fibroblast growth factor
FSH	follicle-stimulating hormone
GnRH	gonadotropin-releasing hormone
GPCR	G protein-coupled receptor
Hh	hedgehog
HSL	homoserine lactone
ICD	intracellular domain
IMD	immune deficiency pathway
JAK	janus kinase
LCO	lipochitooligosaccharide
LH	luteinizing hormone
LRR	leucine-rich repeat
LysM-LK	lysin-motif receptor-like kinase
MAPK	Mitogen-activated protein kinases
MAPKK	Mitogen-activated protein kinases
MAPKKK	Mitogen-activated protein kinases
NO	nitrogen oxide
Nod	nodulation factor
PGN	peptidoglycan
PGRP	peptidoglycan-binding receptor protein
PRR	pattern recognition receptor
PTS	phosphotransferase system
QS	quorum sensing
RLK	receptor-like kinase
RLP	receptor-like protein
TCS	receptor-histidine-kinase two-component system
TNT	tunneling nanotubes
TLR	toll-like receptor
TNF	tumor-necrosis factor
VOC	volatile organic compound
Wnt	vertebrate homolog of drosophila Wingless
